# Coarse-to-Fine Hand–Object Pose Estimation with Interaction-Aware Graph Convolutional Network

**DOI:** 10.3390/s21238092

**Published:** 2021-12-03

**Authors:** Maomao Zhang, Ao Li, Honglei Liu, Minghui Wang

**Affiliations:** School of Information Science and Technology, University of Science and Technology of China, Hefei 230027, China; zmm1@mail.ustc.edu.cn (M.Z.); lhl0796@mail.ustc.edu.cn (H.L.); mhwang@ustc.edu.cn (M.W.)

**Keywords:** hand–object pose estimation, deep learning, graph convolutional network, coarse-to-fine

## Abstract

The analysis of hand–object poses from RGB images is important for understanding and imitating human behavior and acts as a key factor in various applications. In this paper, we propose a novel coarse-to-fine two-stage framework for hand–object pose estimation, which explicitly models hand–object relations in 3D pose refinement rather than in the process of converting 2D poses to 3D poses. Specifically, in the coarse stage, 2D heatmaps of hand and object keypoints are obtained from RGB image and subsequently fed into pose regressor to derive coarse 3D poses. As for the fine stage, an interaction-aware graph convolutional network called InterGCN is introduced to perform pose refinement by fully leveraging the hand–object relations in 3D context. One major challenge in 3D pose refinement lies in the fact that relations between hand and object change dynamically according to different HOI scenarios. In response to this issue, we leverage both general and interaction-specific relation graphs to significantly enhance the capacity of the network to cover variations of HOI scenarios for successful 3D pose refinement. Extensive experiments demonstrate state-of-the-art performance of our approach on benchmark hand–object datasets.

## 1. Introduction

Hand–object interaction (HOI) is a primary part of human daily behavior. Therefore, analyzing hand–object interaction and identifying the poses of hand and object are crucial for understanding and imitating human daily behavior. Recently, hand–object pose estimation has attracted considerable attention in a variety of applications, for example, augmented and virtual reality, robotics, and human–computer interaction.

Although estimating hand pose [1,2,3,4,5,6,7] and object pose [8,9,10,11] in isolation has made remarkable success, jointly estimating both hand and manipulated object poses from a single RGB image remains a challenging task. Particularly, complicated HOI scenarios bring in various issues including not only complex pose variations and self-occlusions that commonly occur in hand-only or object-only pose estimation, but also severe mutual occlusion between hand and manipulated object [12]. Meanwhile, it should also be pointed out that considering the high relevance of hand and manipulated object is helpful for addressing the above issues, since object shape usually enforces physical constraints on hand grasps while hand pose could give cues to object pose and category. Accordingly, there is an urgent need for improving performance in hand–object pose estimation by fully modeling the kinematic relations between hand and object poses in HOI.

In early works [13,14,15], the relations between hand and manipulated object are not taken into account, given the fact that these approaches estimate the poses of hand and object separately. Recently, some works [12,16,17,18,19] start to employ the relations between hand and object as evidence for joint hand–object pose estimation. Despite great progress, most existing approaches try to model hand–object relations during converting 2D poses to 3D poses. However, it is worth noting that generating 3D poses from 2D coordinates is inherently ill-posed problem [20]. In particular, there may exist multiple valid 3D interpretations corresponding to the same 2D representation. In consequence, these methods have limitations in precisely catching and leveraging the relations between hand and object to estimate 3D hand–object poses. Differently, in this work we focus on modeling hand–object relations effectively in the process of refining 3D poses.

To address the above limitations, we propose a novel coarse-to-fine framework for joint hand–object pose estimation, which can progressively improve the performance via multi-step reasoning. In particular, given a monocular RGB image containing hand and object, we first estimate heatmaps of hand joints and object corners in the 2D image, which are then passed through pose regressor to generate coarse 3D poses of hand joints and object corners. After that, an effective graph-based model named Interaction-aware Graph Convolutional Network (InterGCN), is proposed to perform pose refinement by fully leveraging the hand–object relations in 3D context. In this manner, the coarse-to-fine cascade enables our approach to increase the robustness of hand–object pose estimation and achieve more accurate 3D pose results.

Instead of catching hand–object relations during converting 2D poses to 3D poses, we show that in the process of refining coarse 3D poses, the proposed InterGCN can significantly improve performance by explicitly modeling the relations between hand and object. To model rich relations of hand–object interaction, we construct a graph where the nodes are hand joints and object corners. Based on the prior that keypoints have strong relations when they are close to each other, we construct a graph by nearest neighbor analysis on the training dataset, which reflects a general pattern across HOI scenarios. However, the relations between hand and object often change dynamically according to different HOI scenarios, which means that the general graph is insufficient to model complex hand–object relations. To relieve this problem, we propose to learn an additional interaction-specific graph from representations of graph nodes, which delineates a unique pattern of hand–object relations for each HOI scenario. In an effective and specialized way, this interaction-specific graph can capture variations of HOI scenarios for successful 3D pose refinement.

The core contributions of our work are summarized as follows:

- We propose a novel deep learning framework for hand–object pose estimation, which can progressively improve the model’s performance using a coarse-to-fine strategy;
- We introduce Interaction-aware Graph Convolutional Network to explicitly model rich and dynamic hand–object relations to optimize the coarse pose results;
- Extensive experimental results on benchmarks demonstrate that our approach outperforms state-of-the-art methods.

## 2. Related Works

Our work closely relates to joint 3D hand–object pose estimation and graph convolutional networks for dealing with graph-structured data.

### 2.1. Hand–Object Pose Estimation

Early approaches [13,14,15] focus on estimating the pose of hand or object in isolation, which nevertheless may ignore strong relations between hand and manipulated object. Subsequently, by utilizing interaction of hand and object as additional constraints, several works [17,21,22] try to jointly estimate hand and object poses with multi-view RGB or depth input. However, due to various limitations, such as the high cost for a multiple RGB sensors system and the huge power consumption using active depth sensors [12], some researchers shift their focus to RGB-based hand–object pose estimation methods. Several recent works [16,23] employ generative methods, e.g., the MANO [24], to extract geometrical dynamic constraints of hand and object. Nevertheless, such methods usually need extra dense annotations that are usually difficult to obtain in practice.

Given RGB images, there have been some other effective approaches [12,18,19] for hand–object poses estimation using relations between hand joints and object corners. Tekin et al. [18] adopt a unified 3D detection framework to directly output the poses of hand and object without explicitly considering the physical constraints on hand–object interaction. More recently, some methods try to model the hand–object relations during the process of converting 2D poses to 3D. Doosti et al. [19] first regress 2D keypoint locations of hand and object, and then adopt an adaptive graph convolutional network to learn a non-linear mapping between 2D and 3D poses. Instead of using predefined adjacency matrix in graph convolution layer, they use a parameterized matrix learned from training data to capture relations between hand and object. Huang et al. [12] propose a non-autoregressive transformer module to lift 2D poses to 3D where multi-head self-attention layers are employed to model correlations between hand joints and object corners. However, generating 3D poses from 2D coordinates is inherently ill-posed [20] since there may exist multiple valid 3D interpretations for a single 2D representation, which in consequence may cause unreasonable hand–object relations and invalid results. Instead of obtaining relations during the process of lifting 2D poses to 3D, we propose InterGCN to model hand–object relations and leverage them to refine 3D poses.

### 2.2. Graph Convolutional Networks

Graph convolutional networks (GCNs) are introduced to capture inherent dependencies between nodes of graph-structured data. In general, the principle of constructing GCN follows two mainstreams: spectral perspective [25,26] and spatial perspective [27,28,29]. Spectral GCNs use the spectral representations of graphs to perform graph convolution operations. Cai et al. [20] adopt a spectral GCN to handle graphs with predefined and fixed topology for 3D pose estimation, which nevertheless lacks the flexibility and capacity to model latent relations among nodes. In comparison, spatial GCNs directly perform convolution filters on graph vertexes and their neighbors and usually can handle topology-varied graphs. For hand–object pose estimation, Doosti et al. [19] utilize an adaptive GCN to model subtle relationships between joints by constructing a parameterized relation graph. However, this approach tries to learn a general graph for different HOI scenarios, which may be suboptimal for all the samples due to the complexity and variability of HOI in real-world applications. Instead, we propose InterGCN that takes advantages of not only prior knowledge of general hand–object relations, but also interaction-specific relations automatically learned from representations of hand joints and objects corners.

## 3. Methods

**Figure 1:**
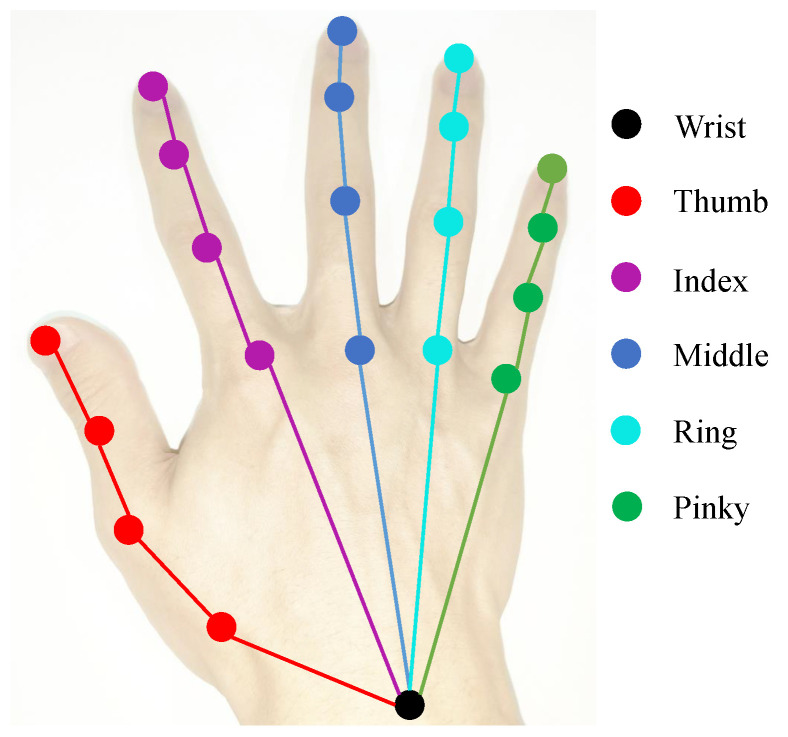
The illustration of hand model with 21 joints.

**Figure 2:**
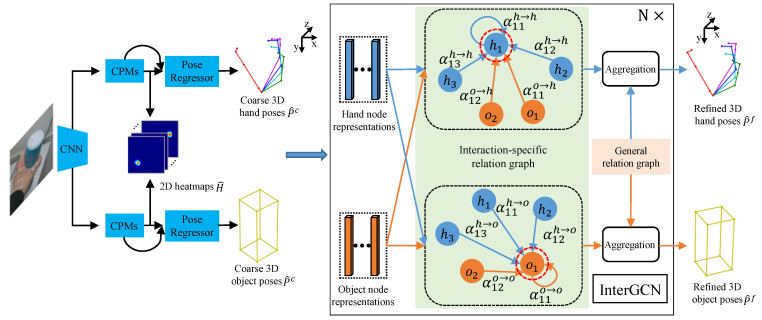
The overview of the proposed coarse-to-fine framework. Our method consists of two stages: coarse stage and fine stage. In the coarse stage, hand and object keypoint heatmaps are first obtained from input image. Subsequently, the heatmaps are passed to the pose regressor to generate coarse 3D hand–object poses. As for the fine stage, InterGCN is introduced to refine coarse hand–object poses estimated in the previous stage by leveraging the general relation graph (Section 3.2.2) and interaction-specific relation graph between hand joints and object corners in 3D context.

Given a single RGB image containing hand and object, our goal is to figure out 3D hand–object poses, where hand pose is defined by a set of joint coordinates $p^{h}={\left(x_{m},y_{m},z_{m}\right)}_{m=1}^{M}$ in 3D space while object pose is represented by a set of bounding box corner coordinates $p^{o}={\left(x_{n},y_{n},z_{n}\right)}_{n=1}^{N}$. *M* and *N* are the number of hand joints and object corners, respectively. In our case, we choose M=21 and N=8 since skeletal model of the hand used in this work has 21 joints and object pose is represented by 8 object bounding box corners. The hand model with 21 joints is shown in Figure 1. The overall scheme of the proposed coarse-to-fine framework is presented in Figure 2. Our method consists of two stages: coarse stage and fine stage. In the coarse stage, image is passed through a 2D pose estimation network to obtain the heatmap of each keypoint, which is used as the input of pose regressor to generate the coarse 3D poses of hand and object. As for the fine stage, InterGCN is proposed to leverage both general hand–object relations and interaction-specific relations learned from representations of hand joints and object corners to refine the predicted coarse poses in 3D context. Details are described in the later sections.

### 3.1. Heatmap-Based Coarse Pose Generation

In this stage, we formulate localization of 2D keypoints as estimation of 2D heatmaps. The 2D heatmap $H_{k}\in R^{H\times W}$ represents the likelihood of the *k*th keypoint at each pixel location, where *H*, *W* denote the height and width of heatmap, respectively. We employ a 2D heatmap estimation network with encoder–decoder architecture based on the Convolutional Pose Machines (CPMs) by Wei et al. [30]. Given image feature representations generated by the encoder, an initial heatmap is predicted and iteratively enhanced in resolution. The number of total iterations are fixed at 3. Furthermore, inspired by [16], we utilize a two-branch network on top of a common CNN encoder to estimate 2D poses of hand and object separately. Note that the two branch networks are different and have independent learnable parameters. Subsequently, a simple yet effective pose regressor similar to [3] is adopted to generate coarse 3D poses of hand and object from the obtained 2D heatmaps. In addition to the generated 2D heatmaps, the intermediate image features in 2D pose estimation network are also fed into the pose regressor by following [31].

### 3.2. Interaction-Aware Graph Convolutional Network

#### 3.2.1. Graph Convolution

In this section, we introduce graph convolution in InterGCN, accounting for the heterogeneous types of nodes.

We first describe a typical Graph Convolutional Network introduced by Kipf and Welling [25]. Let G=(V,E) donate a graph where *V* is a set of |V| nodes and *E* represents the set of edges between the nodes in *V*. Graph convolutional operation can be formulated as:

(1) $$X^{\left(l+1\right)}=\sigma \left(\hat{A}X^{\left(l\right)}W^{\left(l\right)}\right),\hat{A}={\tilde{D}}^{-\frac{1}{2}}\tilde{A}{\tilde{D}}^{-\frac{1}{2}}$$

where $X^{\left(l\right)}\in R^{\left|V\right|\times d^{\left(l\right)}}$ and $X^{\left(l+1\right)}\in R^{\left|V\right|\times d^{\left(l+1\right)}}$ are input and output node representations at *l*th layer with dimensionality of $d^{\left(l\right)}$ and $d^{\left(l+1\right)}$, respectively. $X^{\left(l\right)}$ is a $\left|V\right|\times d^{\left(l\right)}$ input feature matrix. Each row in $X^{\left(l\right)}$ is a $d^{\left(l\right)}$-dimensional vector representing a graph node. $W^{\left(l\right)}\in R^{d^{\left(l\right)}\times d^{\left(l+1\right)}}$ is the learnable weight matrix, σ is activation function (e.g., ReLU). $\tilde{A}=A+I$ is the new self-loop adjacency matrix where $A\in R^{\left|V|\times |V\right|}$ is the adjacency matrix of *G* and *I* is identity matrix. $\tilde{D}$ is the diagonal node degree matrix of $\tilde{A}$. $\hat{A}$ is the symmetric normalized version of $\tilde{A}$. The $\hat{A}$ is a |V|×|V| matrix and represents relations among graph nodes, i.e., the value of ${\hat{A}}_{i,j}$ represents relation between node *i* and node *j*. The edge information in $\hat{A}$ is used to aggregate features in each layer of GCN.

Despite its effectiveness, the direct application of GCN in hand–object pose estimation may limit capability of the network due to node heterogeneity issue. Specifically, there are two different types of nodes in HOI scenarios: hand joints *h* and object corners *o*, which are associated with distinctive pose characteristics as well as feature spaces. To better adapt GCN for hand–object pose estimation, we exploit heterogeneous graph convolution, which takes into account the difference of node types and adopts respective weight matrices for feature transformation. The operation can be represented as:

(2) $$X_{\tau }^{\left(l+1\right)}=\sigma \left(\sum_{\tau^{\prime }\in \Gamma }A_{\tau \tau^{\prime }}X_{\tau^{\prime }}^{\left(l\right)}W_{\tau^{\prime }}^{\left(l\right)}\right)$$

where τ, $\tau^{\prime }\in \Gamma =\left\{h,o\right\}$, $A_{\tau \tau^{\prime }}\in R^{|V_{\tau }\left|\times \right|V_{\tau^{\prime }}|}$ is the submatrix of *A*, with rows and columns denoting the nodes with type τ and their neighboring nodes with type $\tau^{\prime }$, respectively. The node features $X_{\tau }^{\left(l+1\right)}$ are generated by aggregating features of their neighboring nodes $X_{\tau^{\prime }}^{\left(l\right)}$ with different types $\tau^{\prime }$ using different weight matrix $W_{\tau^{\prime }}^{\left(l\right)}\in R^{d^{\left(l\right)}\times d^{\left(l+1\right)}}$. The weight matrix $W_{\tau^{\prime }}^{\left(l\right)}$ takes into account difference of distinct feature spaces and maps them into an implicit common space.

As the core of graph convolution, the adjacency matrix is utilized in InterGCN to explicitly model rich relations of hand and object. Specifically, it consists of two main parts: $A^{g}$ representing general relation graph and $A^{s}$ representing interaction-specific relation graph. In the following sections, we describe how to construct these two graphs and leverage them to update the node representations in detail.

#### 3.2.2. General Relation Graph

To explicitly model hand–object relations, the general relation graph $A^{g}$ is disentangled into non-overlapped subgraphs, i.e., hand-to-hand subgraph $A_{hh}^{g}$, object-to-object subgraph $A_{oo}^{g}$, object-to-hand subgraph $A_{ho}^{g}$ and hand-to-object subgraph $A_{oh}^{g}$. Specifically, the normalized adjacency matrix $A_{hh}^{g}\in R^{M\times M}$ and $A_{oo}^{g}\in R^{N\times N}$ indicate the physical structure of hand skeleton and object bounding box, respectively. Meanwhile, we adopt a data-driven way (Algorithm 1) to build $A_{ho}^{g}$, $A_{oh}^{g}$, based on the prior that keypoints have strong relations when they are close to each other. Specifically, we assemble 3D hand joint coordinates and object corner coordinates of the training dataset into data tensor $P^{h}\in R^{F\times M\times 3}$ and $P^{o}\in R^{F\times N\times 3}$, respectively, where *F* is the number of samples and the last dimension represents the (x,y,z) coordinates. For hand joint *i*, if *j* is the closest object corner to it, then corner *j* is considered as the one most closely related to joint *i* and $A_{ho}^{g,(i,j)}+=1$. Subsequently, we normalize matrix $A_{ho}^{g}$ by scaling the rows so that the row sums are all equal to one. With these operations, we encode general object-to-hand relations in $A_{ho}^{g}$. That is, $A_{ho}^{g,(i,j)}$ represents the relative importance of the object corner *j* to the hand joint *i*. In the same way, we can obtain subgraph $A_{oh}^{g}$ that reflects general hand-to-object relations.
**Algorithm 1** Construct relation subgraphs $A_{ho}^{g}$, $A_{oh}^{g}$**Input:** 3D hand pose labels $P^{h}\in R^{F\times M\times 3}$, 3D object pose labels $P^{o}\in R^{F\times N\times 3}$, *F* is sample number of training dataset.**Output:** relation matrix $A_{ho}^{g}\in R^{M\times N}$, $A_{oh}^{g}\in R^{N\times M}$.1:Initialize $A_{ho}^{g}$, $A_{oh}^{g}$ with zeros.2:**for** each pose $\left(p^{h},p^{o}\right)\in \left(P^{h},P^{o}\right)$ **do**3:   **for** i=0 to *M* **do**4:     $j^{*}=\underset{j}{argmin}\;{∥p_{i}^{h}-p_{j}^{o}∥}_{2}$5:     $A_{ho}^{g,(i,j^{*})}+=1$6:   **end for**7:   **for** i=0 to *N* **do**8:     $j^{*}=\underset{j}{argmin}\;{∥p_{i}^{o}-p_{j}^{h}∥}_{2}$9:     $A_{oh}^{g,(i,j^{*})}+=1$10:   **end for**11:**end for**

#### 3.2.3. Interaction-Specific Relation Graph

Graph $A^{g}$ implies the relations between the hand joints and the object corners. Nevertheless, it only accounts for a general pattern across HOI scenarios, which may not be optimal for all the samples. For example, in HOI scenario “closing juice bottle”, the relations between fingertips and bottle cap should be stronger, but it is not true for some other scenarios, such as “pouring juice bottle” and “putting salt”. This fact suggests that hand–object relations should be interaction-specific and data dependent, which unfortunately is not supported in graph $A^{g}$.

**Figure 3:**
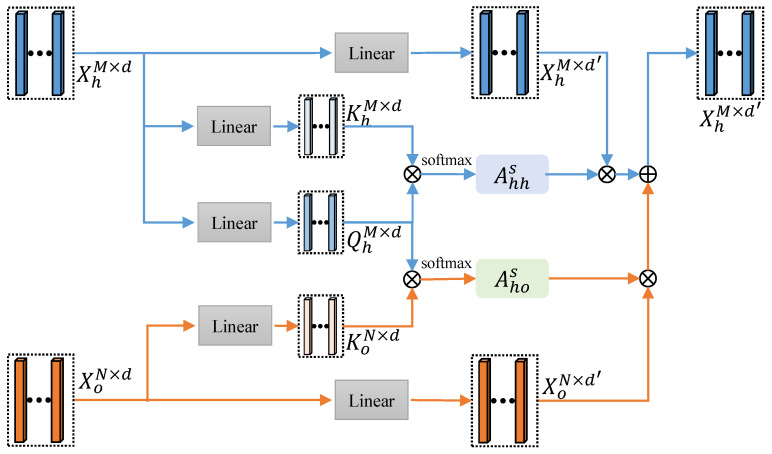
Implementation details of hand-centric attention mechanism and feature aggregation. General relation graph $A^{g}$ is not shown here for clarity.

To solve this issue, we further present an interaction-specific relation graph $A^{s}$ which is unique for each HOI scenario with hand- and object-centric attention mechanism. As shown in Figure 3, given representations of hand joints and object corners $X_{h}\in R^{M\times d}$, $X_{o}\in R^{N\times d}$, three linear layers are applied to generate query vector $Q_{h}\in R^{M\times d}$ for hand joints, and two key vectors: $K_{h}\in R^{M\times d}$ and $K_{o}\in R^{N\times d}$ for hand joints and object corners, respectively. They are then multiplied to obtain a M×M hand-to-hand subgraph $A_{hh}^{s}$ and a M×N object-to-hand subgraph $A_{ho}^{s}$. $A_{hh}^{s,(i,j)}$ and $A_{ho}^{s,(i,j)}$ indicate the impact of node *j* with type *h* and node *j* with type *o* to node *i* with type *h*, respectively. The value of subgraphs is normalized to 0–1, which is used as the soft edge of two nodes. With a softmax operation, we can calculate $A_{hh}^{s}$ and $A_{ho}^{s}$ as follows:

(3) $$\begin{array}{cc} A_{hh}^{s,(i,j)} & =\frac{\exp(X_{h}^{i}W_{h}^{Q}{\left(X_{h}^{j}W_{h}^{K}\right)}^{T})}{\sum_{j=1}^{M}\exp\left(X_{h}^{i}W_{h}^{Q}{\left(X_{h}^{j}W_{h}^{K}\right)}^{T}\right)}\\ A_{ho}^{s,(i,j)} & =\frac{\exp(X_{h}^{i}W_{h}^{Q}{\left(X_{o}^{j}W_{o}^{K}\right)}^{T})}{\sum_{j=1}^{N}\exp\left(X_{h}^{i}W_{h}^{Q}{\left(X_{o}^{j}W_{o}^{K}\right)}^{T}\right)} \end{array}$$

where $W_{h}^{Q}$, $W_{h}^{K}$ and $W_{o}^{K}$ are the parameters of the linear layers for computing the hand query $Q_{h}$, hand key $K_{h}$ and object key $K_{o}$. $X_{h}^{i}$, $X_{h}^{j}$, and $X_{o}^{j}$ are node features. In the same manner, we can construct object-to-object subgraph $A_{oo}^{s}$ and hand-to-object subgraph $A_{oh}^{s}$ with object-centric attention mechanism.

#### 3.2.4. Feature Aggregation

With the general and the interaction-specific relation graphs, we can update the representations of hand joints and object corners by aggregating features of other graph nodes. As shown in Figure 3, considering the distinct node types in InterGCN, two parallel linear layers are applied to encode features of hand joints and object corners, respectively. After that, we aggregate features from not only hand joints but also object corners by leveraging both general relation graph and interaction-specific relation graph. This process is computed by replacing Equation (2) with the following layer-wise propagation rule:

(4) $$X_{h}^{\left(l+1\right)}=\sigma \left(\left(A_{hh}^{g}+A_{hh}^{s}\right)X_{h}^{\left(l\right)}W_{h}^{\left(l\right)}+\left(A_{ho}^{g}+A_{ho}^{s}\right)X_{o}^{\left(l\right)}W_{o}^{\left(l\right)}\right)$$

Similarly, we can update the representations of object corners as:

(5) $$X_{o}^{\left(l+1\right)}=\sigma \left(\left(A_{oo}^{g}+A_{oo}^{s}\right)X_{o}^{\left(l\right)}W_{o}^{\left(l\right)}+\left(A_{oh}^{g}+A_{oh}^{s}\right)X_{h}^{\left(l\right)}W_{h}^{\left(l\right)}\right)$$

### 3.3. Loss Functions

We train our model with three loss functions: heatmap loss $L_{hm}$, coarse 3D pose loss $L_{c}$, and refined 3D pose loss $L_{f}$.

#### 3.3.1. Heatmap Loss

$L_{hm}=\sum_{j=1}^{M+N}{∥H_{j}-{\hat{H}}_{j}∥}_{2}^{2}$, where $H_{j}$ and ${\hat{H}}_{j}$ denote ground-truth and estimated heatmap, respectively. We set the heatmap resolution as 32×32 pixels. The ground-truth heatmap is defined as 2D Gaussian with standard deviation of 1 pixel centered on the ground-truth 2D location.

#### 3.3.2. 3D Pose Loss

Three-dimensional pose loss composes of two parts: coarse pose loss $L_{c}=\sum_{j=1}^{M+N}{∥P_{j}-{\hat{P}}_{j}^{c}∥}_{2}^{2}$ and refined pose loss $L_{f}=\sum_{j=1}^{M+N}{∥P_{j}-{\hat{P}}_{j}^{f}∥}_{2}^{2}$, where $P_{j}$, ${\hat{P}}_{j}^{c}$ and ${\hat{P}}_{j}^{f}$ denote ground-truth, coarse pose and refined pose with InterGCN, respectively.

The total loss function for training is:

(6) $$L=L_{hm}+\alpha L_{c}+\beta L_{f}$$

where α=1, and β=2 are weight coefficients to balance different loss functions.

## 4. Experiments

### 4.1. Datasets

First-Person Hand Action (FPHA) Dataset [32] is a large-scale and commonly used dataset for 3D hand–object pose estimation, containing 1175 videos of hand actions performed by 6 subjects from egocentric point-of-view. Three-dimensional hand poses are obtained automatically with visible magnetic sensors. A subset of FPHA contains object pose labels for 4 objects (juice bottle, liquid soap, milk, and salt) involving a variety of action categories, which is denoted as FPHA-HO and adopted here for performance evaluation. Briefly, position and orientation with 6 degrees of freedom are obtained using six magnetic sensors attached to subject’s hand. Then, hand pose is derived from inverse kinematics of a defined hand model. As for object pose, one more sensor is attached to the closest point to the center of mass.

HO-3D [33] is a recently released hand–object dataset, which consists of video sequences of hands interacting with objects from third-person point-of-view. HO-3D is a markerless hand–object dataset containing 77 k annotated frames, 65 sequences, 10 subjects, and 10 objects. Then, 66 k frames with 3D hand–object pose labels are used as training set. Note that hands are only annotated with the wrist coordinates in testing set containing 11 k frames.

ObMan [16] is a synthetic dataset containing 141 k training images, 6 k validation images, and 6 k testing images. Images in this dataset are obtained by rendering 3D hand meshes with 8 objects from ShapeNet [34]. We pretrain the proposed model on this synthetic dataset, and then fine-tune on above two datasets.

### 4.2. Evaluation Metrics

Following [12], mean error in Euclidean space between estimated joint coordinates and ground truth is used to evaluate 3D hand poses. The percentage of correct keypoints (3D PCK) is also adopted to measure accuracy of hand pose estimation. For evaluating object pose, we utilize the percentage of correct poses (PCP). In this metric, if the 2D projection error of object corners is less than a given threshold, object pose is considered to be estimated correctly. Moreover, we also provide the area under the curve (AUC) on the PCK for hand pose and the PCP for object pose. Euclidean distance is used to calculate 2D and 3D error. We use mean error to determine PCP and PCK.

### 4.3. Implementation Details

Using a resized 256×256 HOI image as input, the 2D pose estimation network generates keypoint heatmaps with resolution 32×32. We adopt ResNet-18 [35] as the encoder of 2D pose estimation network. Instead of training the whole network together, we first train the 2D pose estimation network and the pose regressor for 60 epochs with an initial learning rate of 0.01 and multiply it by 0.1 every 20 epochs. Then, we optimize InterGCN for another 60 epochs, starting from a learning rate of 0.01 with a shrink factor of 0.1 every 20 epochs. Finally, we fine-tune the whole network for 40 epochs, and the learning rate starts from 0.001 and is multiplied by 0.1 every 20 epochs. We use Adam as network optimizer and set the batch size to 64. The hyperparameters are selected based on grid search strategy over validation dataset. Specifically, the initial learning rate is selected from {0.1,0.01,0.001,0.0001} and batch size is selected from {16,32,64,128}. All experiments are conducted on GeForce GTX 1080Ti GPU using PyTorch framework. For both FPHA-HO [32] and HO-3D [33] datasets, we use official train and test splits, and pretrain on ObMan [16].

### 4.4. Comparisons with the State-of-the-Arts

In this section, we report the performance of our approach and compare with the state-of-the-art methods. Similar to [12], the absolute 3D poses in camera coordinate system (c.s.) are denoted as Abs. while the root-relative 3D poses are represented as Rel. Hand pose and object pose are denoted as HP and OP, respectively.

**Figure 4:**
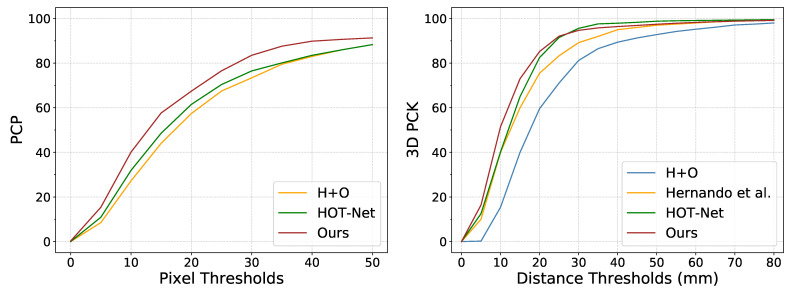
Comparison with state-of-the-art methods on PCP curve and 3D PCK curve.

We first compare the proposed method with others on FPHA-HO [32] using the mean 3D distance error of hand and object. As shown in Table 1, our approach compares favorably with the state-of-the-art approaches H+O [18] and HOT-Net [12], especially for the relative hand pose (8.32 vs. 10.41). In terms of the PCP metric of object pose, as illustrated in Figure 4, our approach outperforms H+O and HOT-Net by a large margin over all the thresholds. For 3D PCK curve of hand pose, we compare with H+O and HOT-Net as well as another depth-based method [32]. The results in Figure 4 demonstrate the promising performance of the proposed method.

The Area Under Curve (AUC) scores on the PCK curve for hand pose and the PCP curve for object pose are also reported on FPHA-HO [32] and HO-3D [33]. Since hands in the testing dataset are only labeled with wrist coordinates (without complete hand pose annotations), the AUC on PCK for HO-3D are just for wrist keypoint. As seen in Table 2, the AUC score of our method reaches 0.839 for hand pose and 0.654 for object pose on FPHA-HO. Compared to HOT-Net [12], the proposed method achieves 1.21% and 9.91% gains, which indicates the effectiveness of our method. Even on less constrained and more complex dataset HO-3D, our light weight model remains competitive against HOT-Net [12].

### 4.5. Ablation Study

In this section, FPHA-HO [32] is used to identify the effects of the components of our method.

#### 4.5.1. Effectiveness of Coarse-to-Fine Strategy

This self-comparison experiment is carried out to show the significant improvement by adopting the coarse-to-fine strategy in hand–object pose estimation. “Ours Coarse” means the coarse 3D pose results without using InterGCN for refinement. As shown in Table 1, compared to “Ours Coarse”, our full coarse-to-fine model remarkably reduces the mean 3D distance error by 1.28 mm (from 16.25 to 14.97), 1.72 mm (from 10.04 to 8.32), and 4.24 mm (from 27.31 to 23.07) for Abs. HP, Rel. HP, and Abs. OP, respectively. Obviously, the results of this contrast experiment prove that coarse-to-fine cascade can significantly boost the performance of hand–object pose estimation.

#### 4.5.2. Effectiveness of Relation Graphs

**Figure 5:**
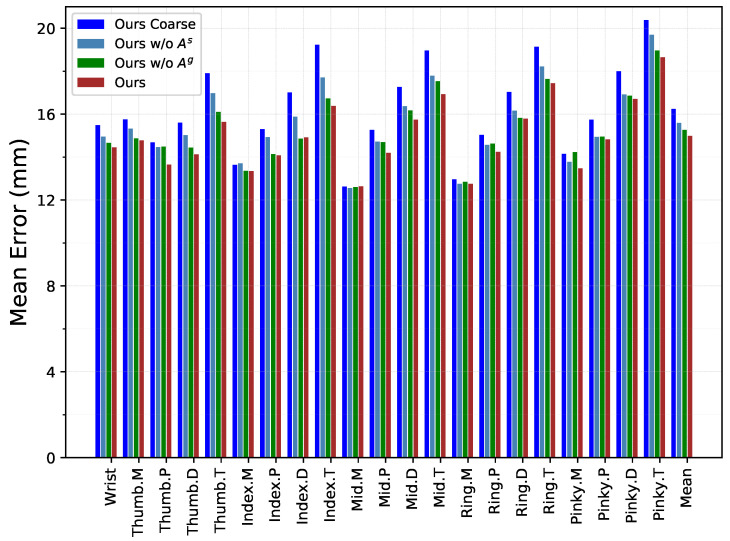
Three-dimensional distance error per hand joints. Note that M, P, and D denote the 3 consecutive joints located between the wrist and fingertip (T) on each finger, in their order.

As introduced in the method section, there are two types of graphs representing hand–object relations in each layer of InterGCN, i.e., general relation graph $A^{g}$ and interaction-specific relation graph $A^{s}$. To examine the effect of using different relation graphs for hand–object pose estimation, two sets of experiments are conducted. As presented in Table 3, the results show that the two relation graphs are both beneficial for hand–object pose estimation and that deleting any one of the two graphs will degrade the performance. It is also worth noting that removing interaction-specific relation graph leads to a significantly greater decline in performance, demonstrating that modeling unique relations for each HOI scenario plays a crucial role in hand–object pose estimation. With all relation graphs added together, the model achieves the best performance. In addition, Figure 5 breaks out the distance error of each hand joint, which is in line with the above results and further illustrates the effectiveness of relation graphs.

#### 4.5.3. Visualization of Relation Graphs

**Figure 6:**
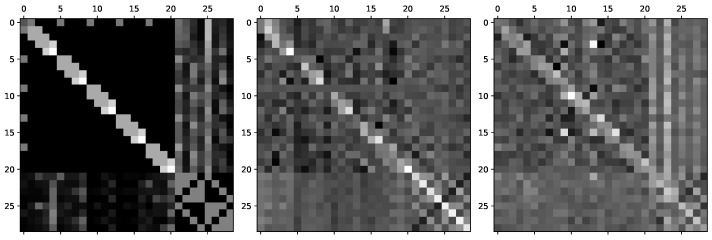
Visualization of the general relation graph and the learned interaction-specific relation graphs in different HOI scenarios. The left matrix is the general relation graph in the FPHA-HO dataset. The middle matrix is an example of the interaction-specific relation graph learned by our InterGCN in “pouring juice bottle”. The right matrix is another example of the interaction-specific relation graph for “closing juice bottle”.

Figure 6 displays the general relation graph in the FPHA-HO dataset and two examples of the interaction-specific relation graph learned by InterGCN in two different HOI scenarios: “pouring juice bottle”, and “closing juice bottle”. The gray scale of each element in the matrix represents the strength of the relation between two nodes. From the right matrix, we can see from the columns that some object corners (node 21, 23) have stronger effect on the fingertips (e.g., node 4, 8, 12) in “closing juice bottle”. Such relations does not seem to exist in “pouring juice bottle”, as illustrated in the middle matrix. This difference shows that the learned interaction-specific graph can explicitly model unique relations between hand and object for different HOI cases.

### 4.6. Qualitative Results

**Figure 7:**
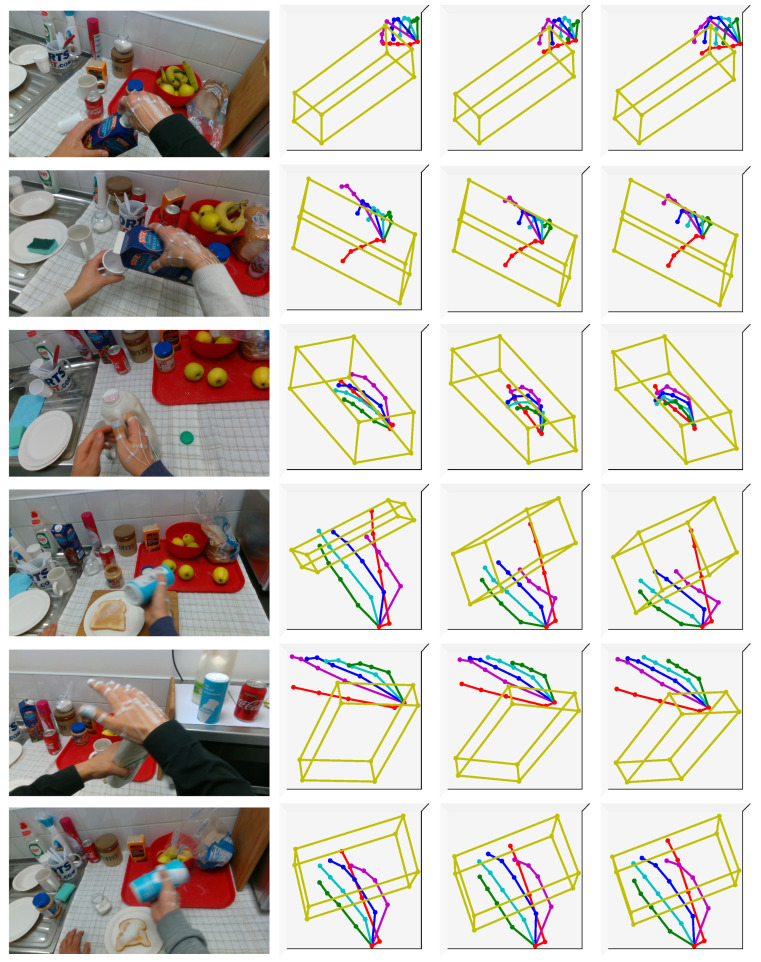
Qualitative results of our method on the FPHA-HO dataset. From left to right: input image, coarse pose results without using InterGCN, refined pose results, and ground truth. The proposed coarse-to-fine framework can handle diverse HOI scenarios with different objects and severe occlusions.

Some qualitative results of our method on the FPHA-HO are illustrated in Figure 7, which also show the effectiveness of coarse-to-fine framework. It is clearly that more reliable results can be consistently achieved in the fine stage, despite of the complexity and versatility of hand and object poses. For example, from the visualized coarse 3D pose results in the first row, it can be seen that the thumb and index fingers are pinched together, which is incorrect for the HOI scenario “closing juice bottle”. In comparison, the visualized refined 3D poses suggest that the thumb and index fingers correctly make a “C” shape to grasp and tighten the juice bottle cap, which is remarkably consistent with the ground truth of hand poses. Taken together, these results further suggest that our method has a strong capability in estimating hand–object poses for different HOI scenarios.

### 4.7. Runtime

In this section, we report training and inference time of our model. Our model is easy to train. It takes about 18 and 40 h to train the proposed coarse-to-fine framework on FPHA-HO and HO-3D using a GeForce GTX 1080Ti GPU, respectively. As for inference time, it takes about 0.02 s to estimate hand–object poses from a single sample image.

## 5. Conclusions

In this paper, a novel deep-learning framework is proposed to jointly estimate hand–object poses, which can progressively improve the performance with coarse-to-fine strategy. We focus on refining the coarse poses with InterGCN by modeling and leveraging the rich and dynamic relations between the hand and the manipulated object. In addition to a general relation graph across HOI scenarios, we also propose to utilize an interaction-specific relation graph to catch unique pattern for each interaction sample. With this manner, our method can effectively increase the capacity of the network to cover variations of HOI scenarios for effective 3D pose refinement. We carry out extensive experiments and detailed ablation studies to show the superiority of our method. In the future, we will try to evaluate person-independent hand pose estimation with customized training and testing data spilt.

## Figures and Tables

**Table 1:** Comparison with state-of-the-art methods on FPHA-HO. The mean 3D distance error (mm) is used as metric (Lower is better).

Model	Abs. HP Error	Rel. HP Error	Abs. OP Error
H+O	15.81	-	24.89
HOT-Net	15.18	10.41	21.37
Ours Coarse	16.25	10.04	27.31
Ours	14.97	8.32	23.07

**Table 2:** Comparison with state-of-the-art methods on both FPHA-HO and HO-3D using the AUC scores (Higher is better) on 3D PCK curve and PCP curve.

Dataset	Model	AUC on PCK	AUC on PCP
FPHA-HO	HOT-Net	0.829	0.595
	Ours	0.839	0.654
HO-3D	HOT-Net	0.819	0.567
	Ours	0.805	0.583

**Table 3:** Comparison of mean 3D distance error (mm) when using InterGCN without general relation graph $A^{g}$ or interaction-specific relation graph $A^{s}$ on FPHA-HO.

Method	Abs. HP Error	Rel. HP Error	Abs. OP Error
Ours Coarse	16.25	10.04	27.31
Ours w/o $A^{s}$	15.59	9.14	24.83
Ours w/o $A^{g}$	15.26	8.79	23.89
Ours	14.97	8.32	23.07

## Data Availability

Data available in a publicly accessible repository. Data citation: [dataset] Garcia-Hernando, G.; Yuan, S.; Baek, S.; Kim, T.-K. 2018. First-Person Hand Action (FPHA) Dataset; https://guiggh.github.io/publications/first-person-hands (accessed on 2 July 2020); DOI: 10.1109/CVPR.2018.00050; [dataset] Hampali, S.; Rad, M.; Oberweger, M.; Lepetit, V. 2020. HO-3D; https://www.tugraz.at/index.php?id=40231 (accessed on 26 August 2020); Version 2; DOI: 10.1109/CVPR42600.2020.00326; [dataset] Hasson, Y.; Varol, G.; Tzionas, D.; Kalevatykh, I.; Black, M.J.; Laptev, I.; Schmid, C. 2019. ObMan; https://hassony2.github.io/obman (accessed on 22 July 2020); DOI: 10.1109/CVPR.2019.01208.
